# Simulation Study: The Impact of Structural Variations on the Characteristics of a Buried-Channel-Array Transistor (BCAT) in DRAM

**DOI:** 10.3390/mi13091476

**Published:** 2022-09-05

**Authors:** Minjae Sun, Hyoung Won Baac, Changhwan Shin

**Affiliations:** 1 Department of Semiconductor Display Engineering, Sungkyunkwan University, Suwon 16419, Korea; 2 Samsung Institute of Technology, Samsung Co., Ltd., Hwaseong 18448, Korea; 3 Department of Electrical and Computer Engineering, Sungkyunkwan University, Suwon 16419, Korea; 4 School of Electrical Engineering, Korea University, Seoul 02841, Korea

**Keywords:** buried channel array transistor, structural variation, short channel effects, device characteristics

## Abstract

As the physical dimensions of cell transistors in dynamic random-access memory (DRAM) have been aggressively scaled down, buried-channel-array transistors (BCATs) have been adopted in industry to suppress short channel effects and to achieve a better performance. In very aggressively scaled-down BCATs, the impact of structural variations on the electrical characteristics can be more significant than expected. Using a technology computer-aided design (TCAD) tool, the structural variations in BCAT (e.g., the aspect ratio of the BCAT recess-to-gate length, BCAT depth, junction depth, fin width, and fin fillet radius) were simulated to enable a quantitative understanding of its impact on the device characteristics, such as the input/output characteristics, threshold voltage, subthreshold swing, on-/off-current ratio, and drain-induced barrier lowering. This work paves the road for the design of a variation-robust BCAT.

## 1. Introduction

The physical dimensions of transistors in integrated circuit (IC) have been scaled down (i) to make the density of the transistors in ICs as high as possible and (ii) to improve the electrical performance of the transistors [1,2]. However, as the channel length of the transistors (including the metal oxide semiconductor field effect transistor (MOSFET)) has been aggressively decreased, and the short channel effect (SCE) has adversely affected the devices’ performance. Note that the threshold voltage in MOSFET becomes lower with a shorter channel length (which is an undesirable secondary effect). To address the SCE issue, many engineering solutions have been proposed, such as (i) the use of a new device architecture to enhance the gate-to-channel capacitive coupling (e.g., fin-shaped FET, ultra-thin-body FET, and multiple-bridge-channel FET), (ii) the use of novel materials (e.g., SiGe in source/drain or compound semiconductors in the channel to induce appropriate stress, resulting in better mobility of the electrons/holes), and (iii) the high-k/metal-gate technique for achieving an electrically thin but physically thick gate oxide layer [3,4,5,6,7,8]. One of the solutions for overcoming the SCE in DRAM (dynamic random-access memory) cell transistors is to adopt the buried-channel-array transistor (BCAT) structure. This device structure can increase the effective channel length due primarily to its recessed channel and buried gate structure [9]. However, as the 3D physical dimensions of BCATs have been significantly scaled down, it is important to study the impact of structural variations on the electrical characteristics of BCATs. Moreover, process-induced systematic/random variation results in undesired alterations to the device characteristics [10]. Thus, the impact of structural variations on the electrical characteristics of BCATs must be quantitatively studied in order to control device characteristics in mass production and pave the road for the design of next-generation BCAT devices. In this work, a BCAT device with 20 nm-long gate length was simulated with a 3D technology computer-aided design (TCAD) tool (i.e., Synopsys Sentaurus). To obtain a nominal device structure, its device design parameters were altered in order to reveal the ways in which each parameter’s variation affected the device characteristics.

## 2. Baseline Device Design and Its Structural Variations

**Figure 1:**
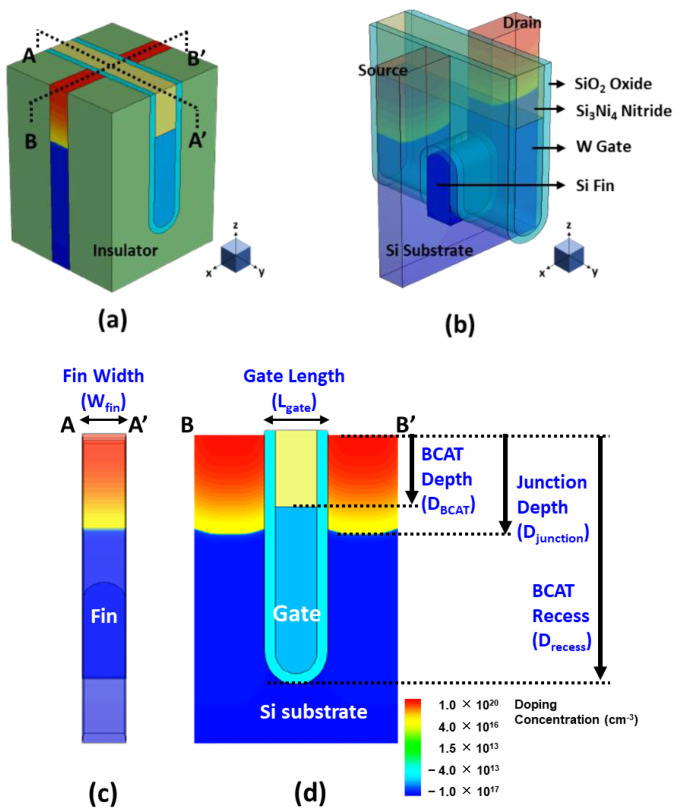
(**a**) A 3D bird’s eye view of the buried-channel-array transistor (BCAT), (**b**) its saddle-fin-shaped silicon channel, and its cross-sectional view (**c**) across the channel (A–A’) and (**d**) along the channel (B–B’). Note that the substrate/body region was doped with 10^17^ cm^−3^ boron, while the source and drain regions were counter-doped with 10^20^ cm^−3^ arsenic.

**Figure 2:**
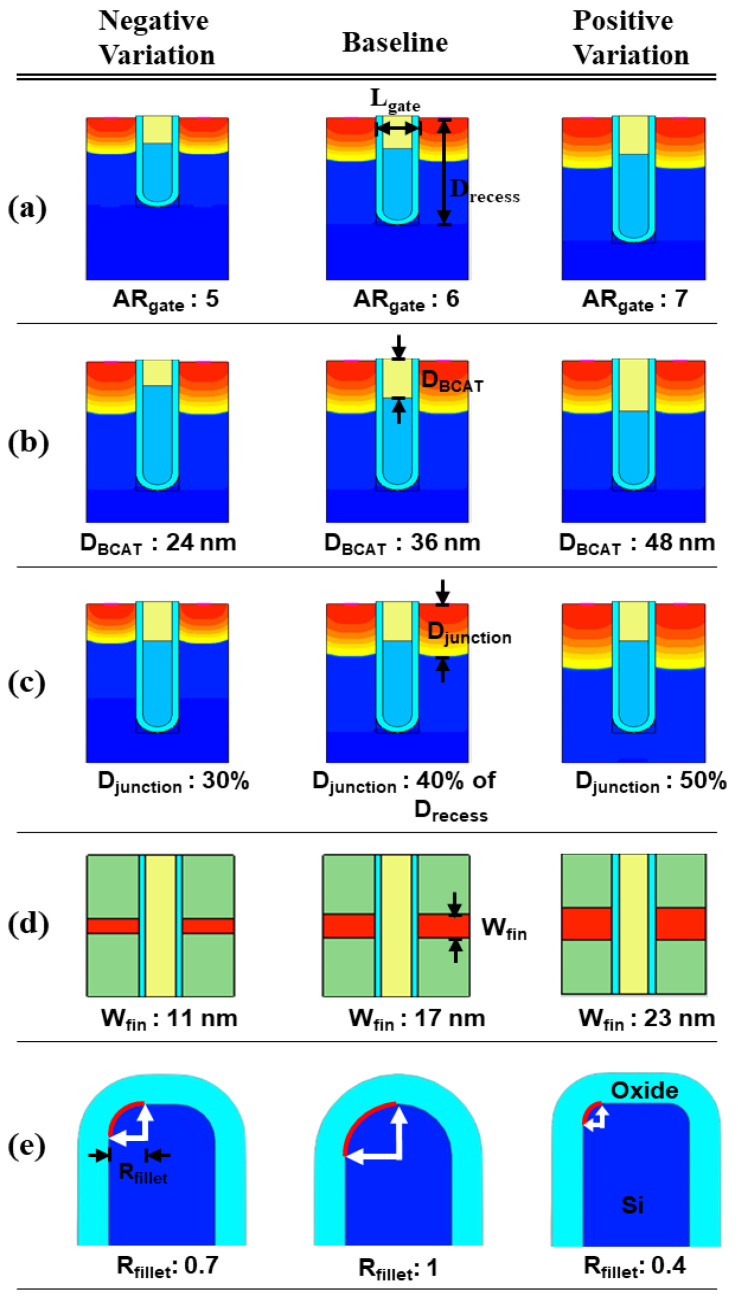
Parametric variations in the BCAT: (**a**) aspect ratio of the BCAT recess (D_recess_)-to-gate length (L_gate_) (i.e., AR_Gate_ = D_recess_/L_gate_), (**b**) BCAT depth (D_BCAT_), (**c**) junction depth (D_junction_), (**d**) fin width (W_fin_), and (**e**) fin fillet radius (R_fillet_).

The BCAT device was built with the Sentaurus TCAD tool, and a 3D bird’s eye view of the device is shown in Figure 1a. The saddle-fin-shaped silicon channel was buried under the nitride (Si_3_Ni_4_) insulator layer and covered by the tungsten gate and silicon oxide (see Figure 1b). The cross-sectional views across the channel and along the channel is shown in Figure 1c,d, respectively. The physical gate length (L_gate_) and recess (D_recess_) of the baseline BCAT device was nominally set to 20 nm and 120 nm, respectively, resulting in a D_recess_/L_gate_ (=AR_gate_) of ~ 6.0 (see Figure 2a). Its gate material was tungsten, with the working function of 4.8 eV. The recessed region and the saddle fin of the baseline BCAT device were surrounded by the gate oxide of 5 nm. Note that the D_BCAT_ corresponded to the thickness of the nitride in gate stack (see Figure 2b). The silicon substrate/body region was doped with 10^17^ cm^−3^ boron, while the source and drain regions were counter-doped with 10^20^ cm^−3^ arsenic. Note that the Gaussian doping profile was used for the device. The nominal junction depth (D_junction_) was set to 40% of D_recess_ (see Figure 2c vs. Figure 2a). The saddle fin width (W_fin_) of the nominal BCAT device was set to 17 nm (see Figure 2d). The saddle fin fillet radius (R_fillet_) was defined as the multiplication factor of the saddle fin radius (see Figure 2e). This quantitatively indicated whether the saddle fin shape was rounded or angled [11]. The nominal R_fillet_ was set to 1.0 to ensure that the saddle fin was shaped as a semi-circle.

The impacts of structural variations in baseline device on its electrical characteristics were investigated. The aspect ratio of D_recess_ to L_gate_ (i.e., AR_gate_) varied from 5 (−17% of the baseline) up to 7 (+17% of the baseline). Note that the AR_gate_ of the baseline device structure was 6. The BCAT depth (D_BCAT_) varied from 24 nm (−33% of the baseline) to 48 nm (+33% of the baseline). Note that the D_BCAT_ of the baseline device was 36 nm. The D_junction_ (which is defined as the depth at which the doping concentration is 10^17^ cm^−3^) varied from 30% to 50% of the D_recess_. The saddle fin width (W_fin_) varied from 11 nm to 23 nm (i.e., −33 ~ +33% of the baseline value). Note that the nominal saddle fin width (W_fin_) of the baseline device was 17 nm. The saddle fin fillet radius (R_fillet_) of the baseline device was 1.0, and it varied from 0.4 (−60% of the baseline) to 0.7 (−30% of the baseline). For each structural variation mentioned above, the input/output characteristics (i.e., I_D_-vs.-V_G_/I_D_-vs.-V_D_) were simulated (see Table 1).

The Philips unified mobility model was adopted for the simulations to assess the dependence of the mobility on the electron-hole scatterings, screening of ionized impurities by charged carriers, and clustering of impurities [12]. The mobility degradation at the semiconductor–insulator interfaces due to surface roughness scattering was incorporated into in the simulations using the Lombardi mobility model [13]. The Canali model was taken into account for the carrier velocity saturation in the regions with high and low electric fields [14]. The Hurkx trap-assisted tunnelling model was used to observe the band-to-band tunnelling [15].

## 3. Results and Discussion

For the given parameters, including AR_gate_, D_BCAT_, D_junction_, W_fin_, and R_fillet_, the input/output characteristics (i.e., I_D_-vs.-V_G_/I_D_-vs.-V_D_) of the baseline device were simulated with the structural variations. The results are summarized in Table 1.

**Figure 3:**
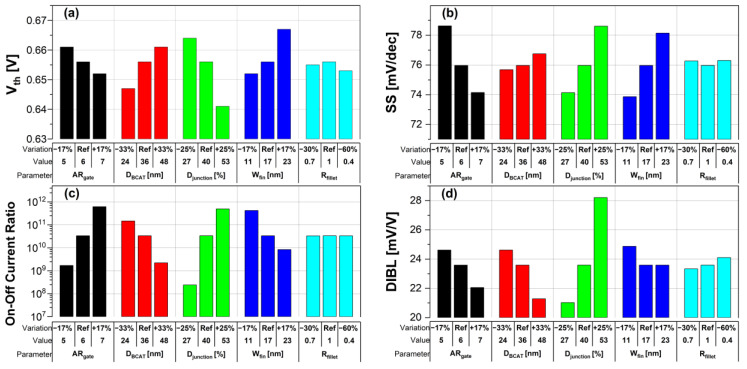
Performance metrics of BCAT: (**a**) threshold voltage (V_th_), (**b**) subthreshold swing (SS), (**c**) on-/off-current ratio, and (**d**) drain-induced barrier lowering (DIBL) for given parametric variations.

It is noteworthy that the short channel effects of the DRAM cell transistor in the ~ 20 nm technology mode were effectively rebuilt (see the I_D_ vs. V_D_ plots in Table 1). From the I_D_ vs. V_G_ plots, key device characteristics were extracted, and they are summarized in Figure 3a–d. Herein, the threshold voltage (V_th_) was defined using the constant current method (i.e., the constant current = 10^−7^ A × W (channel width)/L (channel length) [16]). The nominal (baseline structure) device performance metrics were: V_th_ = 0.656 V; SS = 76 mV/dec; on-/off-current ratio = 3.4 × 10^10^; and DIBL = 23.6 mV/V. Note that these results are within reasonable ranges (V_th_ ≈ 0.7 V; SS < 90 mV/dec; on-/off-current ratio ≈ 10^10^; DIBL ≈<50 mV/V) compared to the other reported/published experimental results, such as those of the 20 nm FinFET device or 30 nm buried-word-line-structured device [17,18]. Higher AR_gate_ induced a lower V_t__h_, steeper subthreshold swing (SS), better drain-induced barrier lowering (DIBL), and a higher on-/off-current ratio. This is due primarily to the higher height of the saddle fin, as well as the channel region being more closely surrounded by the gate, resulting in a higher gate-to-channel coupling capacitance. Note that increasing AR_gate_ significantly affected the SS (i.e., from 74.1 mV/dec to 78.6 mV/dec; nominal SS = 76.0 mV/dec) and on-/off-current ratio (i.e., from 2.2 × 10^9^ to 6.2 × 10^11^; nominal on-/off-current ratio = 3.4 × 10^10^) at ± 17% of the baseline. Though increasing the AR_gate_ yielded a better performance in terms of the device characteristics (i.e., a steeper SS, higher on-/off-current ratio, and lower DIBL), the difficulties involved in the fabrication process must be considered. To achieve the higher AR_gate_, a deeper BCAT recess (D_recess_) at the same gate length is needed, requiring more advanced technologies in the lithography and etching processes. In addition, the increase in AR_gate_ must be limited to the marginal point in order to avoid the risks of the bending/leaning of the Si active substrate or voids in the gate materials (tungsten, in this study) during the deposition process (due to imperfect deposition in the deep BCAT recessed area) [19,20]. Increasing the D_BCAT_ resulted in a lower V_th_, non-steeper SS, lower on-/off-current ratio, and a worse DIBL. This is mainly because (1) the gate controllability decreased as the effective gate length became shorter (herein, the effective gate length was defined as the distance from the bottom of the nitride layer at the source side to that at the drain side), and because the (2) electric field intensity of the metal gate became weak. In regard to the fabrication process flow, the parameter D_BCAT_ can be controlled by adjusting the amount of the metal gate etch-back. If the metal gate etch-back or cleaning process is stable in the process deviation, it may provide a useful option for achieving the higher SS, on-/off- current, and lower DIBL [21].

However, the purpose of the stacking nitride insulator layer on top of the metal gate is to reduce gate-induced drain leakage (GIDL) by isolating the metal gate and drain and reducing the metal gate–drain overlapped region. Thus, the D_BCAT_ must be adjusted and limited to the appropriate level in order to meet the device specifications, such as the GIDL. Increasing the D_junction_ resulted in lower V_th_, worse DIBL, and non-steeper SS. This is due to the shortening of the effective channel length with the increasing D_junction_ [22]. Varying the D_junction_ might yield a better device performance without changing the physical dimensions of the device; thus, it has the advantages of avoiding any undesired defections caused by the process/structure. It was shown that increasing the D_junction_ (i.e., −33% to +33% of baseline) resulted in remarkable decrease in the V_th_ (from 0.664 V to 0.641 V; nominal V_th_ = 0.656 V) and increase in the on-/off-current ratio (from 2.4 × 10^8^ to 5.0 × 10^11^; nominal on/off-current ratio = 3.4 × 10^10^). However, as the DIBL increases from 21.0 mV/V to 28.2 mV/V, caused by the SCE, the control of the D_junction_ must be carefully considered by compensating for the SCE (i.e., silicon-on-insulator or junction engineering, such as pocket implanting, etc.). A narrower fin width (W_fin_) resulted in a lower V_th_, non-steeper SS, and higher on-/off-current ratio. The channel region becomes fully depleted with the narrower fin width, so that the gate controllability of the channel region is enhanced [23]. A narrower W_fin_ would require more advanced technologies in the fabrication process (i.e., smaller-scale lithography or etching processes), as the lateral size of the active Si becomes smaller. Similar to those of the AR_gate_, the risks of the leaning/bending of the active silicon substrate must be considered, since the aspect ratio of the height of the active silicon to W_fin_ increases as the W_fin_ decreases. Compared to the other parametric variations, the parameter, R_fillet_, resulted in the least significant variation (i.e., <5%) in the device performance. This is because the saddle fin width was less than the fin height (W_fin_ = 17 nm, H_fin_ = 48 nm). Otherwise, the R_fillet_ would have affected the device performance. If the corner of the saddle fin becomes more rounded, the device reliability will be less degraded due to the less concentrated electric field at the corner [24,25].

## 4. Conclusions

The buried-channel-array transistor (BCAT) with a 20 nm-long gate length was simulated with the Sentaurus TCAD tool, and then the impacts of the structure variations on its device characteristics were investigated. For the given baseline device structure, the structure parameters including AR_gate_, D_BCAT_, D_junction_, W_fin_, and R_fillet_ were adjusted in order to quantitatively observe the variations in the input/output characteristics and key device performance metrics (i.e., V_th_, SS, on-/off-current ratio, and DIBL). When the D_junction_ was altered (i.e., ±33%), the V_th_ variation (between 0.664 V and 0.641 V; nominal V_th_ = 0.656 V) and DIBL (from 21.0 mV/V to 28.2 mV/V; nominal DIBL = 23.6 mV/V) were most significantly degraded/affected. When the AR_gate_ was increased by +17% (vs. the baseline), the on-/off-current ratio was most significantly increased up to 6.2 × 10^11^ (note that the on-/off-current ratio of the baseline was 3.4 × 10^10^). Among the other structural parameters, it turned out that R_fillet_ minimally affected the device performance (i.e., <5%). Those structural variations, in the end, affected the gate-to-channel capacitances, effective channel length, and depletion regions of the BCAT.

## Figures and Tables

**Table 1:** I_D_-V_G_ and I_D_-V_D_ of the BCAT for given parametric variations, including AR_gate_, D_BCAT_, D_junction_, W_fin_, and R_fillet_. Note that the drain current is normalized to the channel width.

Plot	I_D_-V_G_	I_D_-V_D_
**AR_gate_**	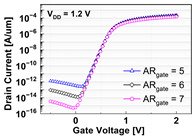	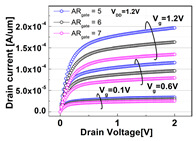
**D_BCAT_**	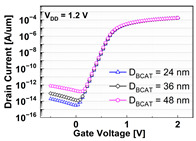	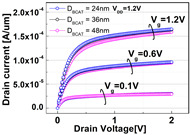
**D_junction_**	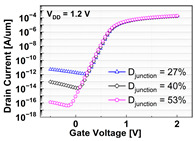	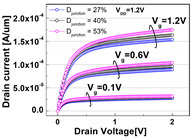
**W_fin_**	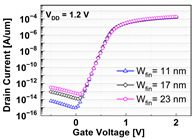	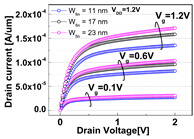
**R_fillet_**	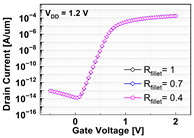	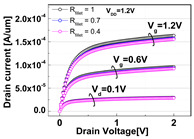

## Data Availability

The data presented in this study are available on request from the corresponding author.
